# Supplementary material for: Monoubiquitination by the human Fanconi anemia core complex clamps FANCI:FANCD2 on DNA in filamentous arrays
Source: eLife. 2020 Mar 13;9:e54128. doi: 10.7554/eLife.54128 (PMC7156235; doi:10.7554/eLife.54128)
Supplement: Supplementary file 1. [file elife-54128-supp1.docx]

Supplementary file 1A. DNA oligonucleotides used in this study. The following oligonucleotides were ordered from IDTDNA.

| **Oligo No.** | **Sequence** |
| --- | --- |
| XOm1 | 5’-ACGCTGCCGAATTCTACCAGTGCCTTGCTAGGACATCTTTGCC  CACCTGCAGGTTCACCC-3' |
| XOm4 | 5’-CGATAGTCGGATCCTCTAGACAGCTCCATGTAGCAAGGCACTGGTAGA  ATTCGGCAGCGT-3’ |
| XOm2.1/2 | 5’-GGGTGAACCTGCAGGTGGGCAAAGATGTCC-3’ |
| XOm3.1/2 | 5’-CATGGAGCTGTCTAGAGGATCCGACTATCG-3’ |
| XOm1.comp | 5'-GGGTGAACCTGCAGGTGGGCAAAGATGTCCTAGCAAGGCACTGGTAGAATTCG  GCAGCGT-3' |
| oligo1-150 | 5'-TAAATAAGATAAGGATAATACAAAATAAGTAAATGAATAAACAGAGAA  AATAAAGTAAAGGATATAAAAAATGAACATAAAGAATAAGTAAATGAATA  AAACATAATAGGAATAAATATAGGAAATGAAATAAAAGAGACATAAATAAGA-3' |
| oligo2-150 | 5'-TCTTATTTATGTCTCTTTTATTTCATTTCCTATATTTATTCCTATTATGTTTTA  TTCATTTACTTATTCTTTATGTTCATTTTTTATATCCTTTACTTTATTTTCTCTGT  TTATTCATTTACTTATTTTGTATTATCCTTATCTTATTTA-3' |

Supplementary file 1B. Combination of oligonucleotides annealed to generate DNA substrates used in this study

| Substrate | Labelled oligo | Mix with cold oligo |
| --- | --- | --- |
| ssDNA | 12 µM X0m1 | - |
| dsDNA | 12 µM X0m1 | 36 µM XOm1.com |
| Splayed arm | 12 µM X0m1 | 36 µM XOm4 |
| 3’ flap | 12 µM X0m1 | 36 µM XOm3.1/2  36 µM XOm4 |
| 5’ flap | 12 µM X0m1 | 36 µM XOm2.1/2  36 µM XOm4 |
| Replication fork | 12 µM X0m1 | 36 µM XOm2.1/2  36 µM XOm3.1/2  36 µM XOm4 |
| 150 bp dsDNA | - | 12 µM oligo1-150  12 µM oligo2-150 |
